# Sam2bam: High-Performance Framework for NGS Data Preprocessing Tools

**DOI:** 10.1371/journal.pone.0167100

**Published:** 2016-11-18

**Authors:** Takeshi Ogasawara, Yinhe Cheng, Tzy-Hwa Kathy Tzeng

**Affiliations:** 1 IBM Research—Tokyo, Tokyo, Japan; 2 IBM Systems, Austin, TX, United States of America; 3 IBM Systems, Poughkeepsie, NY, United States of America; National Centre for Biological Sciences, INDIA

## Abstract

This paper introduces a high-throughput software tool framework called *sam2bam* that enables users to significantly speed up pre-processing for next-generation sequencing data. The sam2bam is especially efficient on single-node multi-core large-memory systems. It can reduce the runtime of data pre-processing in marking duplicate reads on a single node system by 156–186x compared with de facto standard tools. The sam2bam consists of parallel software components that can fully utilize multiple processors, available memory, high-bandwidth storage, and hardware compression accelerators, if available. The sam2bam provides file format conversion between well-known genome file formats, from SAM to BAM, as a basic feature. Additional features such as analyzing, filtering, and converting input data are provided by using *plug-in* tools, e.g., duplicate marking, which can be attached to sam2bam at runtime. We demonstrated that sam2bam could significantly reduce the runtime of next generation sequencing (NGS) data pre-processing from about two hours to about one minute for a whole-exome data set on a 16-core single-node system using up to 130 GB of memory. The sam2bam could reduce the runtime of NGS data pre-processing from about 20 hours to about nine minutes for a whole-genome sequencing data set on the same system using up to 711 GB of memory.

## Introduction

The rapid advance of sequencing technology and its falling cost are driving the use of next-generation sequencing (NGS) in a great variety of domains. The volume of data generated by NGS was projected to double every five months [1]. It is highly desirable to be able to quickly process the raw data from a sequencer to the format (e.g. VCF, CNV) that is ready to be integrated with knowledge base and other data sources for further analysis. Typical 50x coverage of whole genome sequencing (WGS) can easily generate up to 500-GB FASTQ files. The data processes on modern computers are still very time-consuming for such huge data sets. The processing time for the Broad’s Genome Analysis Toolkit (GATK) Best Practices pipeline [2] from reference alignment to variant calling can take up to a day or days to finish [3] for typical 50x coverage of WGS data.

Genome data analysis pipelines involve data pre-processing steps before variant calling, which are necessary to achieve accurate variant calling. They scan input data, analyze reads, and filter out reads that can affect accuracy. The pre-processing steps take FASTQ-format [4] files as input and produce compressed binary files in BAM formats [5], which are widely accepted as common file formats to represent aligned sequenced data. The calibrated BAM files are then used in variant discovery to identify sites where the data display variations that are relative to the reference genome.

The pre-processing steps for SAM parsing, sorting, duplicate marking, and BAM file compression can take tens of hours for a WGS SAM file. Their total runtime dominates the whole pre-processing workflow (explained in the next sub-section) and is a clear performance bottleneck. The purpose of sam2bam is to improve the efficiency of this pre-processing through fully utilizing available CPUs and memory.

The overall architecture of the tools should be redesigned so that computer resources are fully utilized to significantly reduce the runtime of such data pre-processing steps (e.g., by 100x). Current major software tools are single- or partially multi-threaded. Partially multi-threaded tools usually have to wait for data generated from single-threaded components because every component (single- or multi-threaded) is executed one by one. Therefore, they can not fully utilize multiple CPUs that are available at all times. Single-threaded components become bottlenecks in performance on multi-CPU systems. For example, let us assume that 80% of the runtime is executed by multi-threads and the remaining time is executed by a single thread. Speed-up for such tools by using multiple CPUs is limited to 5x even if hundreds of CPUs are available on the system.

The sam2bam simultaneously executes functional components (e.g., file I/O, SAM parsing, and data compression) to achieve further speed-ups on such many-CPU systems, instead of executing the components one-by-one in a large loop. The components are combined as a pipeline. In addition, most steps are multi-threaded. An appropriate number of CPUs are allocated to each component so that no components become a bottleneck in the pipeline. The sam2bam can achieve more than 100x speed-up on a single node system with these redesigned frameworks for NGS data pre-processing.

### Pre-processing Steps for Variant Discovery

Pre-processing steps are necessary to prepare data for analysis to maximize the accuracy of variant discovery. Pre-procesing is also recommended in GATK Best Practices [6]. Pre-processing starts with FASTQ-format files and ends in a calibrated BAM file. Pre-processing for the DNA data usually involves five steps.

1. **Mapping sequence reads to reference genome** This step is usually done by BWA mem [7] or other reference alignment tools and SAM files are generated.
2. **Sorting sequence reads based on coordinates** This step is usually done by Picard SortSam or samtools sort. Some tools that are used in the following steps, such as when the Picard MarkDuplicates tool requires the sorted input files.
3. **Marking duplicate alignments** This step is commonly done by the Picard MarkDuplicates tool to remove the alignments of duplicate reads.
4. **Performing local realignment around indels** This is usually done by GATK RealignerTargetCreator and IndelRealigner tools to reduce artifacts produced in regions around the indels.
5. **Recalibrating the base quality score** This step is usually done by GATK BaseRecalibrator and PrintReads tools to improve the accuracy of base quality scores that the variant calling step relies on.

Preprocessing is very time-cosuming that usually requires tens of hours for a WGS dataset and hours for a whole exome (WEX) dataset. The sorting and duplicate marking steps took most of the runtime for the five steps of preprocessing, which usually range from 60–70% of the total pre-processing time, depending on the tools that were used and the test case size. Therefore, the sorting and duplicate marking steps were identified as a bottleneck in the overall pre-processing steps, and sam2bam was focused on improving the performance of these two steps by redesigning the framework to take advantage of multiple CPU cores, large-capacity memory, and hardware accelerators that are available on modern computers.

## Design and Implementation

**Fig 1:**
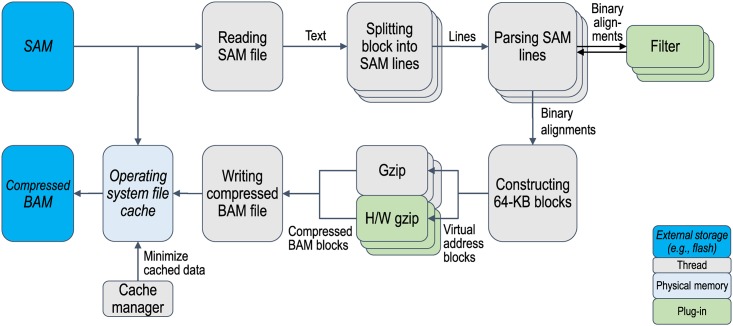
Architecture for sam2bam without analyzer plug-ins. Pipeline is configured with plug-in code that filters out data. Gray boxes indicate steps in pipeline. Steps that have multiple boxes are multi-threaded. Blue boxes denote files in storage. Light-blue boxes denote data in memory. Light green boxes denote plug-in code.

**Fig 2:**
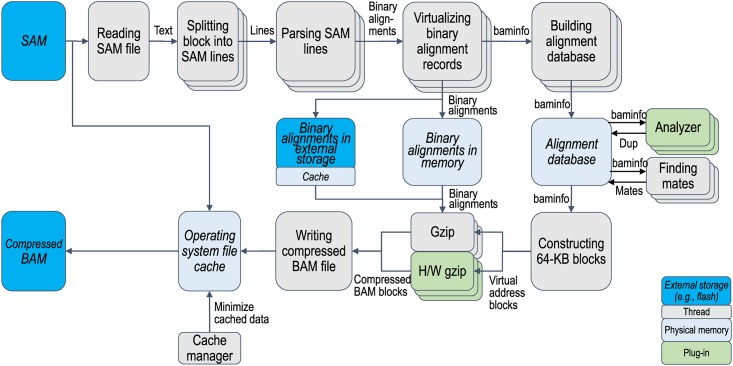
Architecture for sam2bam with analyzer plug-ins. Alignment database is created when analyzer plug-ins are enabled. Binary alignments that are produced by SAM parsing are placed in either main memory or external storage so that they can later be used for generating compressed BAM files by using second half of pipeline. Alignment database has summarized information on binary alignments that is used by analyzer plug-ins.

The main design goal of sam2bam was to provide a high-throughput framework to process genome files at a rate of gigabytes per second (GB/s). The framework consists of a data pipeline that converts the data format from SAM to BAM as outlined in Figs 1 and 2. Data format conversion is divided into multiple steps. Many of these steps are multi-threaded, while a few steps that order the data stream are single-threaded. More CPUs are allocated for more complex steps so that such steps are not bottlenecks in performance. Each step continuously processes data by using CPUs as long as the data are driven from the previous step. While sam2bam provides a data processing pipeline, it uses the samtools/high-throughput sequencing library (HTSLIB) [5] for the data structures and utility functions to handle the SAM and BAM data formats.

### Plug-in Codes

*Plug-in codes* that analyze, filter, and modify data can be attached to sam2bam at runtime. We can develop the plug-in codes and run them on the high-throughput framework. There are three types of plug-in codes.

**Fig 3:**
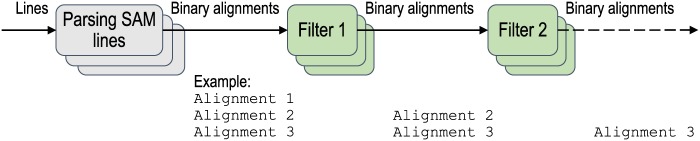
Filter plug-ins. Each filter plug-in transfers binary alignments that meet criteria of filter to go to next step. sam2bam can use multiple filter plug-ins at a time. Each plug-in is executed by multiple threads. In this example, SAM parsing generates three binary alignments. Filter 1 first filters out Alignment 1, and then Filter 2 filters out Alignment 2.

**Fig 4:**
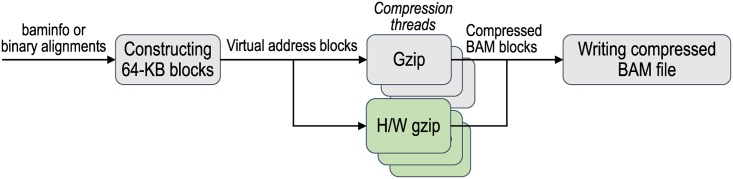
Accelerator plug-in. Compression threads create uncompressed blocks by copying data at virtual addresses. Compression threads that use accelerator plug-in transfer blocks to hardware accelerator, while remaining compression threads compress blocks by using software library code. Compressed blocks are written to BAM file.

**Fig 5:**
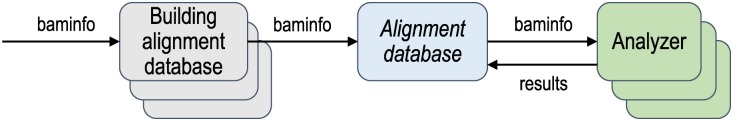
Analyzer plug-ins. Analyzer plug-in codes obtain field values of alignments from database and save analysis results in database. Each analyzer plug-in code can use multiple threads. In this example, three threads execute analyzer plug-in.

- *Filter* The filter plug-in code can be inserted as an additional step of the pipeline (Fig 3). It analyzes input data and determines if the data meet the criteria that the plug-in has. For example, with a filter plug-in that only includes read alignments that overlap a given region of the reference genome, the BAM file that is produced only includes alignments that overlap the specified region.
- *Accelerator* The accelerator plug-in code improves the target pipeline step by using hardware accelerators, such as field-programmable gate arrays (FPGAs). Compression acceleration is currently supported in sam2bam. It produces a compressed BAM file using the standard compression library (or zlib) [8]. If sam2bam detects an accelerator, it automatically offloads compression to hardware while carrying out compression with software (Fig 4). An accelerator that provides the same application programming interface (API) as zlib can be enabled by using the accelerator plug-in.
- *Analyzer* The analyzer plug-in code analyzes a set of read alignments and modifies them. If the analyzer plug-in code is attached to sam2bam, the latter runs the first half of the pipeline that parses data in the SAM format and pools the alignment information in the system. How the alignment information is pooled will be explained in the next subsection (Pipeline Configuration: items 4 and 5). When all alignment information is pooled in the system, the analyzer plug-in scans the pooled information, analyzes it, and generates output on the basis of the analysis (Fig 5). When the analyzer plug-in has completed analysis, the pooled data are transferred to the second half of the pipeline to produce a compressed BAM file. For example, with an analyzer plug-in for marking duplicate alignments, the alignments that were mapped to the same reference region are identified and are marked as duplicates except for the one that has the highest level of quality.

### Pipeline Configuration

The entire process of sam2bam conversion is split into functional stages such as file reading, SAM line detection, and SAM parsing. The stages run in parallel. Each stage can also process multiple data blocks in parallel.

1. **Reading a SAM file** Data blocks (e.g., 64 KB each) are read from a SAM file, which is a sequence of text lines (called SAM lines). Data blocks do not always end at the boundaries between SAM lines. This step adjusts the boundaries between the blocks so that the new blocks end at the SAM line boundaries by scanning each block from the end to the beginning until the first new-line character is found to enable multi-threads to independently process the blocks in the next step.
2. **Splitting a block into SAM lines** SAM lines are extracted from the data blocks transferred from the previous stage. The data blocks are scanned from the beginning to the end to find new-line characters, which separate SAM lines. A major effort in this stage is to find these characters by using a function of the standard C-language library (memchr). The performance of the function is optimized by using vector instructions, if they are available [9]. A scanned data block is transferred to the next pipeline stage, which parses SAM lines with the positions where SAM lines start.
3. **Parsing SAM lines** Binary alignment records are created from the SAM lines. The sam2bam locates a SAM line in the data block at each line position calculated by the previous stage, parses the SAM line by using a library function of Samtools (sam_parse1), and creates a binary alignment record in the data format used in Samtools (bam1_t). The binary alignment record contains the same contents as the corresponding SAM/BAM alignment. Each binary record has a global sequence number, which is created on the basis of the block number and a local number of the record within the block. This global sequence number is used when analysis tools need to know which record has appeared first in the input file for a given set of records.
4. **Virtualizing binary alignment records** The sam2bam supports two modes: all the binary alignment records are placed in the main memory (*memory mode*) or in external storage (*storage mode*). If there is sufficient memory, it is recommended that the memory mode be run to obtain the best performance; otherwise, the storage mode can be used. The binary alignment records are moved to either the main memory or the external storage in this step. The binary alignment records are managed in the later stages in the virtual space of sam2bam. A virtual address is assigned to each binary alignment record. A virtual address can be translated into either a main memory address or an offset of the file in external storage.
  If sam2bam is only invoked with plug-ins that access streaming input data but not pooled information, the input data are transferred from this step to the last step 7. In that case, only the memory mode is enabled. Moreover, steps 5 and 6 are skipped.
5. **Building alignment database** Some plug-ins can start analysis after all the input data are read. An alignment database is built for such plug-ins to provide pooled information on the alignments in this step. Each database entry is represented by a data record called *baminfo*, which is unique to sam2bam. Baminfo is created from each binary alignment record but it only contains information that can be used by the analyzer plug-ins in step 6. Long string data in the binary alignment records are not copied into baminfo. The long string data include reference sequence names, concise idiosyncratic gapped alignment report (CIGAR), sequences, base qualities, and optional fields. If any analyzer plug-in needs any of the omitted data in step 6, the plug-in summarizes the data and saves them into baminfo when baminfo is created.
  The baminfo records are arranged in the reference genome position space to construct the alignment database. The baminfo records can be looked up from the alignment database by using the mapping positions of clipped sequences. The baminfo records that have the same mapping position are gathered and are found in the database by using a single lookup. If there is an analysis that needs the functionality of the baminfo records being looked up by using an unclipped mapping position, the baminfo records are arranged by using the unclipped position as well as the clipped position.
6. **Analyzing baminfo records** The plug-in code undertakes any analysis by using information in the alignment database. Duplicate marking is an example of such code. Multi-threading can accelerate this step by decomposing the input space into sub spaces. For example, we can split the data set of the alignment database into N*B blocks and allocate *B* blocks to each of the *N* threads.
7. **Writing a compressed BAM file** This step groups the binary alignment records into 64-KB blocks (BAM blocks), compresses them, and writes a sequence of the compressed BAM blocks to a BAM file. This step produces a sorted BAM file if the alignment database is available. The sam2bam does not require a separate step for sorting. If some plug-ins set up the alignment database, this step obtains input by scanning the alignment database in the order of sort keys and writes the alignments to the BAM file in the sort-key order. Therefore, all the output from sam2bam is automatically sorted. In contrast, if no analyzer plug-ins are used, the output is not sorted.
  There is a dispatcher that splits a sequence of the binary alignment records into 64-KB sub-sequences by only using their virtual addresses and their sizes. The dispatcher obtains input data by traversing the alignment database or receiving them from step 4. The dispatcher is single-threaded to ensure the order of the binary alignment records in each BAM block is maintained and the order of BAM blocks in the output. It does not construct actual BAM blocks by copying the data to avoid performance bottlenecks in the dispatcher. It instead transfers each set of virtual addresses that construct a single BAM block to a thread pool for compression.
  If the dispatcher receives the input data from step 4, the binary alignment records in the physical memory provide their record sizes. Otherwise, the dispatcher traverses baminfo records in the alignment database to obtain the virtual addresses and record sizes for the binary alignment records.
  Multi-threads are used for compressing BAM blocks to accelerate this step. BAM blocks are constructed and compressed in parallel. The compression method is gzip, which is widely used in the real world [5]. If hardware compression accelerators are available in the system, sam2bam dynamically loads their library codes that can be called via the standard zlib application programming interface (API) and it can offload compression to the accelerators.

## Results and Discussion

To demonstrate that sam2bam can significantly reduce the runtime in marking duplicate alignments, we compared the runtime of sam2bam versus Picard [10], which is a widely-used tool set that is also recommended in GATK best practices [11].

### Experimental Environment

#### Benchmark Data Sets

Two data sets in the SAM format were used to evaluate performance. The first was 150x coverage of WEX data, and second was 50x coverage of WGS data. The WEX data were part of the 1000 Genome Project data [12]. The input SAM file size was 52 GB. The WGS data were part of the Cancer Genome Atlas (TCGA) Benchmark 4 dataset, i.e., G15512.HCC1954.1 [13]. The input SAM file size was 546 GB. The SAM files that we used were created by running the Burrows-Wheeler Aligner (BWA) [7] for the FASTQ-format data converted from the original BAM files.

#### Sam2bam Configuration

The sam2bam handled the SAM and BAM data formats by calling the modified code of samtools. The original code for samtools was obtained from its development repository as of August 2015. Two plug-ins were created for the performance evaluation: an analyzer plug-in for marking duplicate alignments and a compression accelerator plug-in. The source code for sam2bam and the instructions on how to build sam2bam are available from a GitHub repository (https://github.com/t-ogasawara/sam-to-bam).

The analyzer plug-in was enabled to mark duplicate alignments. The sam2bam created the alignment database, as was explained earlier in Section Pipeline Configuration: item 5. The analyzer plug-in traversed the alignment database by using the unclipped position to find the candidates of duplicate alignments and find alignments that had the same beginning and end positions. It also found their pairs by using mate information that is available in the alignment database. The plug-in further used the same criteria as Picard MarkDuplicates [14] for the candidates to select one alignment from duplicates. The duplicates were analyzed in parallel by assigning the segmented unclipped position regions to threads. This analyzer plug-in was provided as a pre-built library for POWER8 systems and was installed when sam2bam was built.

The accelerator plug-in enabled the use of a hardware compression card. Part of the multi-threads for compression offloaded compression tasks to the hardware card instead of performing compression with software. This accelerator plug-in was also constructed when sam2bam was built.

#### Picard Tools

Two Picard tools, SortSam and MarkDuplicates, have been suggested to mark duplicate alignments on GATK Best Practices [11]. SortSam first takes a SAM file as input, sorts the alignments, and writes the result to a BAM file. MarkDuplicates then takes the produced BAM file as the input, marks duplicate alignments, and writes the result to another BAM file. The BAM files are compressed by default.

We used Picard tools (version 2.1.1) [10] in the BioBuilds package (version 2016-04) [15]. OpenJDK 1.8.0_72-internal was used to run the Picard tools with 21 GB of Java heap memory.

#### Hardware

The runtime of the target programs and the maximum size of the memory that was used by the programs during program execution were measured by using a command, /usr/bin/time. The programs were run on a single node of IBM Power Systems S822LC [16] that had 16 POWER8-based CPU cores [17], where 128 logical processors were available (eight logical processors per core) with 1 TB of memory. The machine was attached to high performance storage, i.e., an IBM Elastic Storage Server (ESS) GL4 via a Mellanox FDR switch [18]. A hardware card that provided FPGA-based zlib acceleration [19] was attached to the machine and this could speed up compression of the BAM data. The operating system was Ubuntu 14.04.1.

The theoretical maximum performance for the storage mode of sam2bam that was explained in Pipeline Configuration was measured by using the file system in the main memory (/dev/shm), which simulated an ideal high-performance device (e.g., a solid state drive (SSD)). Such a device is mandatory to achieve high levels of performance in the storage mode since sam2bam in the storage mode performs a huge number of I/O operations that are not always sequential accesses.

### Performance Evaluation with Whole Exome Data

The sam2bam demonstrated more than 100 times better performance than Picard and finished in about one minute in both memory and storage modes while Picard needed more than two hours (Table 1).

**Table 1:** Runtimes and maximum memory sizes for marking duplicates on 52 GB WEX data.

	SAM parsing, sorting	Duplicate marking	Total runtime
Picard	59.4 min (17.5 GB)	86.6 min (22.2 GB)	**146.0 min**
Sam2bam (memory mode)	1.0 min (130.3 GB)		**1.0 min**
Sam2bam (storage mode)	1.0 min (105.4 GB)		**1.0 min**
Sam2bam (memory mode, no HW compression)	1.4 min (129.0 GB)		**1.4 min**
Sam2bam (storage mode, no HW compression)	1.3 min (103.9 GB)		**1.3 min**

Although sam2bam was more than 186 times faster than the standard tools, it required more memory than Picard in the memory mode. The sam2bam reduced the maximum memory size by placing binary alignments in external storage instead of in the main memory in the storage mode. The performance of sam2bam with data compression by using both software and hardware was 43% better than that of sam2bam with software-only compression. The Java heap size for Picard was sufficient since the time spent in garbage collection of the Java heap was negligible (about 0.63% of the total runtime).

The sam2bam benefited from multi-threading and pipelining, which was explained in Pipeline Configuration. The sam2bam read a SAM file for SAM parsing and parsed it at rates of 1.7-2.0 GB/s. This high level of performance was due to multi-threading and pipelining (we will discuss performance without them in S1 Text). The runtime of duplicate marking was 5% of the total runtime.

BAM blocks were compressed at rates of 1.5–1.7 GB/s, including a rate of additional 0.9 GB/s with hardware compression. Such high throughput was achieved by pipelining as well as multi-threading (we will discuss performance without pipelining in S2 Text).

The performance of the framework on which alignments were analyzed and processed is critical for high performance tools. The runtime of a Picard tool that converts the file format from SAM to BAM is 92% that of SortSam and 70% that of MarkDuplicates for Picard (we will discuss the details in S3 Text).

### Performance Evaluation with Whole Genome Sequencing Data

The sam2bam demonstrated 156 times better performance than Picard. The sam2bam finished in about 9 minutes in the memory mode while Picard needed more than 20 hours (Table 2).

**Table 2:** Runtimes and maximum memory sizes for marking duplicates on 546GB WGS data.

	SAM parsing, sorting	Duplicate marking	Total runtime
Picard	631.9 min (18.6 GB)	707.5 min (22.4 GB)	**1339.4 min**
Sam2bam (memory mode)	8.6 min (710.7 GB)		**8.6 min**
Sam2bam (storage mode)	15.6 min (232.9 GB)		**15.6 min**
Sam2bam (memory mode, no HW compression)	16.2 min (709.1 GB)		**16.2 min**
Sam2bam (storage mode, no HW compression)	21.7 min (231.3 GB)		**21.7 min**

The storage mode was 81% slower than the memory mode for WGS data, while the memory and storage modes demonstrated similar performance for WEX data. This slowdown was mainly due to slowdown in BAM block compression in the storage mode (37% of throughput in the memory mode). We collected system-level profiles to analyze the slowdown in BAM block compression. The profiles indicated that the computation time in the operating system was significantly increased by 33 times in the storage mode using the WGS data, but it was only increased by 151% when using the WEX data. We need to further investigate additional activities undertaken by the operating system to address the slowdown with the WGS data in the storage mode.

### Accuracy of Duplicate Marking

The accuracy of duplicate marking for sam2bam could be evaluated by measuring the number of alignments that Picard MarkDuplicates marked but sam2bam did not and also by measuring the number of alignments that sam2bam marked but PicardMarkDuplicates did not. Outputs were compared between Picard MarkDuplicates and sam2bam (we will discuss how we compared the outputs in S4 Text) to evaluate the accuracy of duplicate marking. If sam2bam and Picard MarkDuplicates marked the same sets of alignments, sam2bam could be considered to be accurate and could be used as a fast alternative to Picard MarkDuplicates.

We tested and verified that duplicate marking by sam2bam was *accurate*, based on the experimental results obtained from WEX and WGS data sets. There were 16 million duplicate alignments for the WEX data set and 188 million for the WGS data set. These alignments were the same between sam2bam and Picard MarkDuplicates when making the comparison explained in S4 Text.

## Supporting Information

S1 Text Advantage of multi-threaded and pipelined SAM parsing. (PDF)

S2 Text Evaluation of performance of multi-threaded and pipelined generation of compressed BAM file. (PDF)

S3 Text Discussion on performance of file format conversion framework. (PDF)

S4 Text Methodology of comparing marked duplicates between tools. (PDF)
