# Supplementary material for: Sam2bam: High-Performance Framework for NGS Data Preprocessing Tools
Source: PLoS One. 2016 Nov 18;11(11):e0167100. doi: 10.1371/journal.pone.0167100 (PMC5115855; doi:10.1371/journal.pone.0167100)
Supplement: S1 Text — (PDF) [file pone.0167100.s001.pdf]

S1 Text - Advantage of multi-threaded and pipelined SAM parsing

The sam2bam has high-performance SAM parsing. The performance of SAM parsing can limit the total throughput without the alignment database in sam2bam since sam2bam consists of a single pipeline. With the alignment database, the performance of SAM parsing is critical for the runtime required for data input to the alignment database (e.g., 43% of the total runtime of marking duplicate alignments for WGS data). If SAM parsing is very slow, the total runtime of sam2bam is dominated by the runtime of data input to the alignment database.

The sam2bam was designed to simultaneously parse multiple text lines to achieve high-performance SAM parsing. The sam2bam uses samtool's library code to parse a line text. If a single thread parsed a line text at a time by using the library code, it only provided 8–9% of the SAM parsing rate of sam2bam. Multi-threaded and pipelined SAM parsing is critical to the performance of sam2bam.
