# Supplementary material for: Sam2bam: High-Performance Framework for NGS Data Preprocessing Tools
Source: PLoS One. 2016 Nov 18;11(11):e0167100. doi: 10.1371/journal.pone.0167100 (PMC5115855; doi:10.1371/journal.pone.0167100)
Supplement: S2 Text — (PDF) [file pone.0167100.s002.pdf]

S2 Text - Evaluation of performance of multi-threaded and pipelined generation of compressed BAM file

We evaluated the performance of non-pipelined code by measuring the runtime for generating a compressed BAM file in samtools to evaluate the advantage of the pipelined code that generates a compressed BAM file. An uncompressed BAM file was used to remove the cost of SAM parsing. The standard library that was installed on the operating system (`zlib`) was used to compress BAM blocks on both samtools and sam2bam.

The steps in generating a compressed BAM file are not pipelined in samtools, which has a main loop that sequentially performs three steps: grouping the binary alignments into blocks until a buffer is full, compressing the blocks on the buffer, and writing the compressed blocks to a file. Compressing the blocks is the most time-consuming step, but it can be done by using multi-threads.

The experimental results revealed that the throughput of samtools for generating a compressed BAM file was 38% that of sam2bam without hardware compression. This is because the multi-threads for compression can only run periodically when the buffer is full. The steps in generating a compressed BAM file to continuously run the compression threads should be pipelined so that the threads can compress the blocks without pauses. That is, the compression threads should receive the blocks from a separate thread that groups the alignments into blocks. Also, when they finish compression, they should receive the next blocks without having to wait until the current compressed blocks have been written to a file.
