# Supplementary material for: Sam2bam: High-Performance Framework for NGS Data Preprocessing Tools
Source: PLoS One. 2016 Nov 18;11(11):e0167100. doi: 10.1371/journal.pone.0167100 (PMC5115855; doi:10.1371/journal.pone.0167100)
Supplement: S3 Text — (PDF) [file pone.0167100.s003.pdf]

S3 Text - Discussion on performance of file format conversion framework

The performance of tool frameworks is critical to the total performance of analysis and processing of alignments on tools. The tool frameworks are the software components that read input files, provide data to the tool cores, receive the results from the tool cores, and write the results to output files.

Table 1 summarizes the runtimes of converting the file formats of WEX data from SAM to compressed BAM on Picard and samtools. Compared with the total runtimes of the Picard tools of SortSam and MarkDuplicates (59.4 minutes for the former and 86.6 minutes for the latter), which perform some work in addition to the framework's format conversion, the runtime of the Picard framework (55.2 minutes) is a large part of the total runtime.

The samtools' framework performs 57% better than the Picard's framework on the single-thread configuration. In addition, the performance of the samtools' framework can be improved by accelerating generation of a compressed BAM file by using multi-threads. However, the improvement in performance with multi-threads does not scale well. Sixteen threads on 16 cores only achieved 3.5x speed-up.

Table 1: **Runtimes of file format conversion from SAM to compressed BAM for WEX data.**

**Tool**	**#Threads**	**Runtime**	**Relative runtime**
Picard SamFormatConverter	1	55.2 min	
Samtools view	1	35.2 min	1.00
Samtools view	2	20.6 min	0.59
Samtools view	4	13.6 min	0.39
Samtools view	8	10.5 min	0.30
Samtools view	16	10.0 min	0.28
