# Supplementary material for: Sam2bam: High-Performance Framework for NGS Data Preprocessing Tools
Source: PLoS One. 2016 Nov 18;11(11):e0167100. doi: 10.1371/journal.pone.0167100 (PMC5115855; doi:10.1371/journal.pone.0167100)
Supplement: S4 Text — (PDF) [file pone.0167100.s004.pdf]

# S4 Text - Methodology of comparing marked duplicates between tools

The duplicate marking step marks the alignments of duplicate reads by setting a specific bit of the SAM format to one for the alignments (bit 0x400 of the FLAG field [1]). We first extract alignments whose duplicate bits are one from the output of each tool to compare the outputs between two tools for duplicate marking.

The next step is to normalize the alignments, which involves three substeps: sorting optional fields of the SAM format [1] (e.g., in alphabetical order), removing the optional fields that were added by the tools and are not included in either output, and removing the version of the SAM format.

The last step is file comparison. For example, the `diff` command is available for file comparison in the Linux environment. When sam2bam does not emulate the sorting of Picard SortSam, the order of the marked alignments in the SAM file is also normalized before file comparison so that the same alignment appears in the same position in the files. For example, the `sort` command is available for this normalization in the Linux environment.

The content of the output files produced by the Picard tools and sam2bam included the minor differences that were previously explained, even where the same alignments were marked as duplicates. The Picard MarkDuplicate tool changes the order of the optional fields, while sam2bam retains the original order since it uses the utility functions of samtools, which do not change the order of the optional fields. Also, the Picard MarkDuplicate tool adds an optional field to indicate that MarkDuplicates has processed the file, while sam2bam does not add a MarkDuplicates-specific field. In addition, the Picard MarkDuplicate tool updates the version number in the header of the output, while sam2bam keeps it.

The duplicate marking tools that use the algorithm [2] can find the same set of alignments that include the best alignment and duplicate alignments. The best alignment has the highest base quality while duplicate alignments have base qualities that are lower than the highest base quality. More than a few alignments have the same highest base quality in some cases. Any such alignments can be the best alignment since its base quality is the highest in the alignment set. However, sam2bam chooses the same best alignment from multiple candidates just as Picard MarkDuplicates does by emulating how Picard MarkDuplicates chooses it from an input file that Picard SortSam has sorted. Therefore, the data comparison that was previously explained produces no differences if sam2bam marks appropriate alignments.

# References


[1] The SAM/BAM format specification working group. Sequence Alignment/Map Format Specification. 2015 March 3. Available: http://github.com/samtools/sam-spec.

[2] Overview of the `dedup` function of `bamUtil`. Available: http://genome.sph.umich.edu/wiki/BamUtil:_dedup.
